# JCPyV infection of primary choroid plexus epithelial cells reduces expression of critical junctional proteins and increases expression of barrier disrupting inflammatory cytokines

**DOI:** 10.1128/spectrum.00628-24

**Published:** 2024-06-14

**Authors:** Sheila A. Haley, Bethany A. O'Hara, Christoph Schorl, Walter J. Atwood

**Affiliations:** 1 Department of Cell Biology, Biochemistry, and Molecular Biology, The Warren Alpert Medical School, Brown University, Providence, Rhode Island, USA; University of Wisconsin-Madison, Madison, Wisconsin, USA

**Keywords:** polyomavirus, blood-CSF barrier, choroid plexus, gene expression, junctional proteins, chemokines, PML, neurological disease, neuroinvasion

## Abstract

The human polyomavirus, JCPyV, establishes a lifelong persistent infection in the peripheral organs of a majority of the human population worldwide. Patients who are immunocompromised due to underlying infections, cancer, or to immunomodulatory treatments for autoimmune disease are at risk for developing progressive multifocal leukoencephalopathy (PML) when the virus invades the CNS and infects macroglial cells in the brain parenchyma. It is not yet known how the virus enters the CNS to cause disease. The blood-choroid plexus barrier is a potential site of virus invasion as the cells that make up this barrier are known to be infected with virus both *in vivo* and *in vitro*. To understand the effects of virus infection on these cells we challenged primary human choroid plexus epithelial cells with JCPyV and profiled changes in host gene expression. We found that viral infection induced the expression of proinflammatory chemokines and downregulated junctional proteins essential for maintaining blood-CSF and blood-brain barrier function. These data contribute to our understanding of how JCPyV infection of the choroid plexus can modulate the host cell response to neuroinvasive pathogens.

**IMPORTANCE:**

The human polyomavirus, JCPyV, causes a rapidly progressing demyelinating disease in the CNS of patients whose immune systems are compromised. JCPyV infection has been demonstrated in the choroid plexus both *in vivo* and *in vitro* and this highly vascularized organ may be important in viral invasion of brain parenchyma. Our data show that infection of primary choroid plexus epithelial cells results in increased expression of pro-inflammatory chemokines and downregulation of critical junctional proteins that maintain the blood-CSF barrier. These data have direct implications for mechanisms used by JCPyV to invade the CNS and cause neurological disease.

## INTRODUCTION

The neuroinvasive human polyomavirus JCPyV is a small, nonenveloped, double stranded DNA virus (1–3). Infection is widespread, with seroprevalence ranging from 60 to 80% in populations worldwide (4–6). Typically, the virus establishes a benign, asymptomatic persistent infection in peripheral organs including the kidney and other tissues of the urinary tract (7–9). However, in highly immunosuppressed patients, such as those with HIV/AIDS, hematologic malignancies, or treated with immunomodulatory therapies, the virus can reactivate and cause the often fatal demyelinating disease, progressive multifocal leukoencephalopathy (PML) (10–12). Following the loss of normal immune surveillance of the central nervous system (CNS), the virus invades brain parenchyma, and causes the lytic destruction of oligodendrocytes, leading to demyelination and poor patient prognosis (13). The sole treatment for PML relies on the restoration of immune function that can lead to a damaging inflammatory response called immune reconstitution inflammatory syndrome (IRIS) and worsening neurological symptoms if not managed appropriately. A critical gap in our understanding of JCPyV pathogenesis is that it is not known how the virus overcomes the barriers that restrict the access of pathogens to the CNS.

The CNS is protected from the periphery by specialized structures including the blood brain barrier (BBB) and the blood-CSF barrier (BCSFB). The BCSFB, or choroid plexus, is composed of a core of loosely packed stromal tissue that contains resident immune cells and a fenestrated vasculature (14, 15). This core is surrounded by the choroid plexus epithelium, a specialized monolayer of polarized cells that are bound by tight junctions and create the functional barrier between the CNS and the peripheral blood.

However, the BCSFB is known to be used as a selective entry point to the CNS by both immune cells and neuroinvasive pathogens (16–18). Samples of choroid plexus from patients with PML and JCPyV-associated meningitis have shown productive JCPyV infection (19, 20). It is known that choroid plexus epithelial cells express known protein and carbohydrate entry receptors for JCPyV (21). *In vitro* experiments in primary human choroid plexus cells have also shown virus receptor expression and productive infection (22–24).

To further understand the consequences of virus infection of choroid plexus epithelial cells we profiled genes and proteins that are modulated following virus infection. We found that infection triggered the downregulation of tight junction proteins and the upregulation of inflammatory molecules, both of which are associated with the disruption of the BCSFB.

## RESULTS

### Gene expression changes following infection of primary choroid plexus epithelial cells with JCPyV

**Fig 1:**
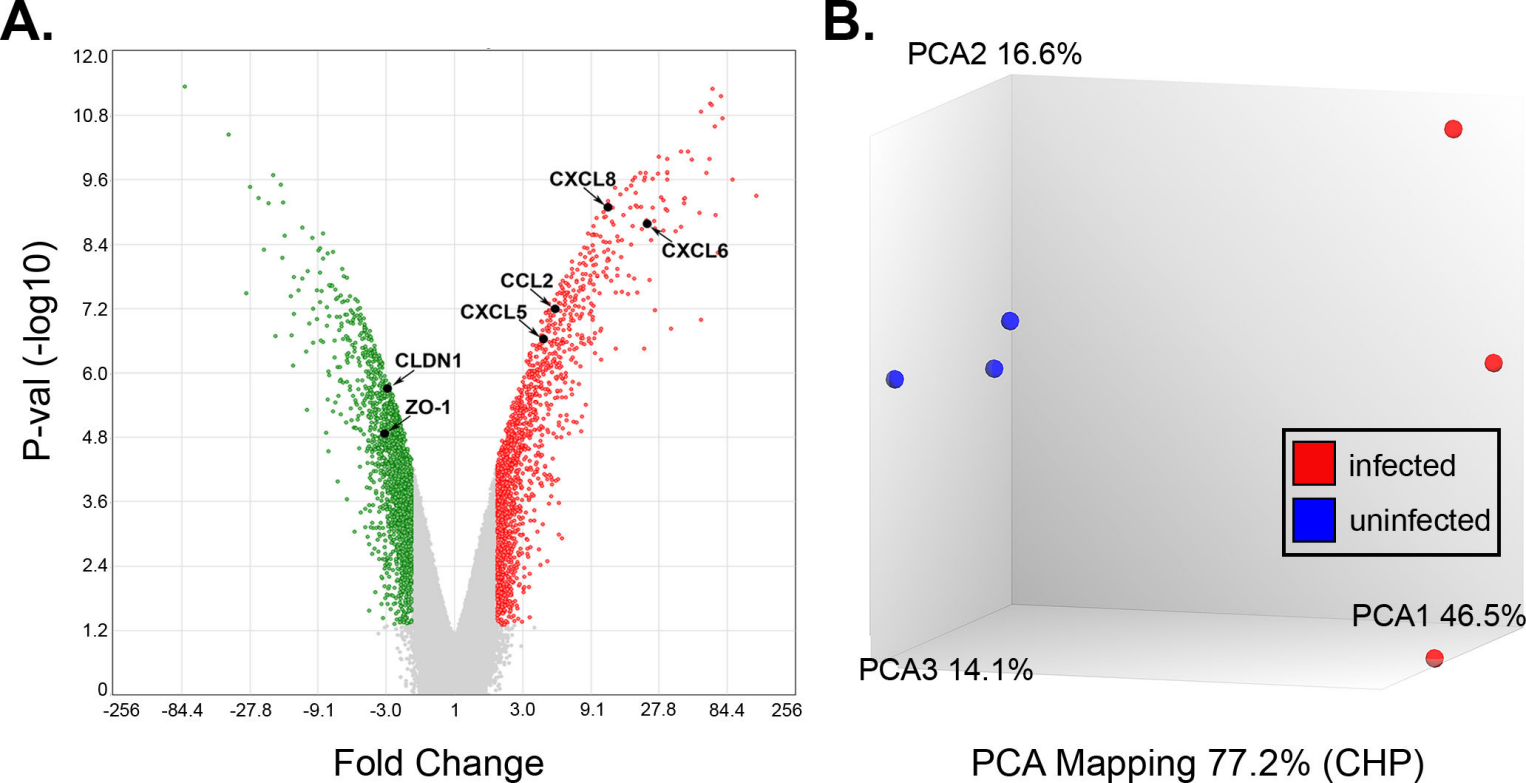
JCPyV infection alters host gene expression in CPEP cells. (**A**) Volcano plot illustrating differentially expressed genes in JCPyV infected (7 d.p.i.) compared to uninfected CPEP cells. Genes with a fold change of >2 for upregulated genes (red) and <−2 for downregulated genes (green) and a false discovery rate (FDR) of *P* < 0.05 were considered differentially regulated. Genes in gray are not significantly changed. (**B**) PCA plot for demonstrating separation of between uninfected (blue) versus infected (red) samples.

Global transcriptome changes as measured by microarray analysis was performed on mRNA isolated from three biological replicates of primary choroid plexus epithelial cells (CPEP) cells infected with JCPyV compared to uninfected controls. After 7 days post-infection (7 d.p.i), distinct differences were observed between the infected and uninfected cells, using a ±2-fold expression cutoff and a significance level of *P* < 0.05. A volcano plot was used to illustrate changes in gene expression following infection with virus (Fig. 1A). Principal component analysis (PCA) of the uninfected vs infected cells showed that gene expression profiles were significantly different from each other and grouped into two separate clusters (Fig. 1B). In our analysis of differentially expressed protein-expressing genes, 382 were upregulated and 580 were downregulated following virus infection.

To better understand how the host cell responds to JCPyV infection, we performed gene ontology (GO) enrichment analysis on significantly changed genes and grouped transcripts into categories according to their biological processes. Regulated genes corresponded to a variety of gene ontologies that included pathways involved in immune signaling, DNA replication and repair, the cell cycle, and cellular adhesion.

### JCPyV infection induces expression and secretion of chemokines

**Fig 2:**
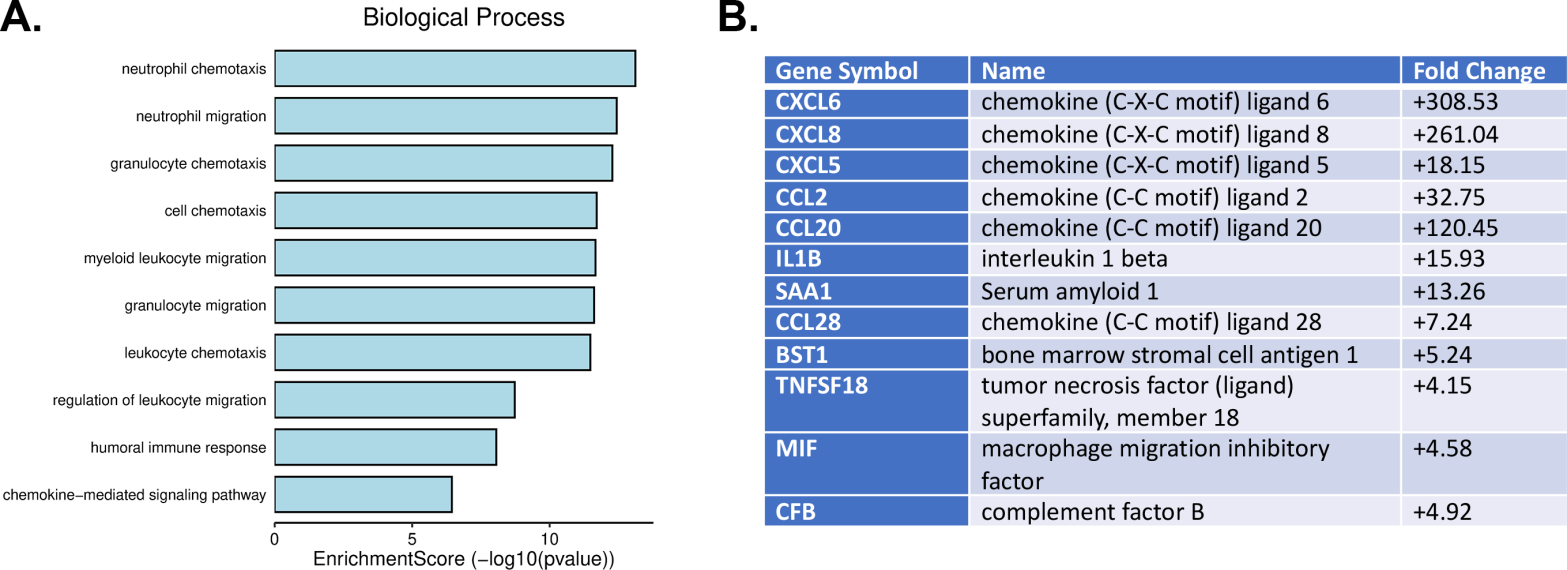
Expression of proinflammatory genes increases following JCPyV infection. (**A**) GO term analysis of genes upregulated by JCPyV infection following the filtering of cell division/DNA replication processes (described in Materials and Methods). (**B**) Fold changes of highly regulated genes contributing to the regulation of biological functions in panel **A**.

**Fig 3:**
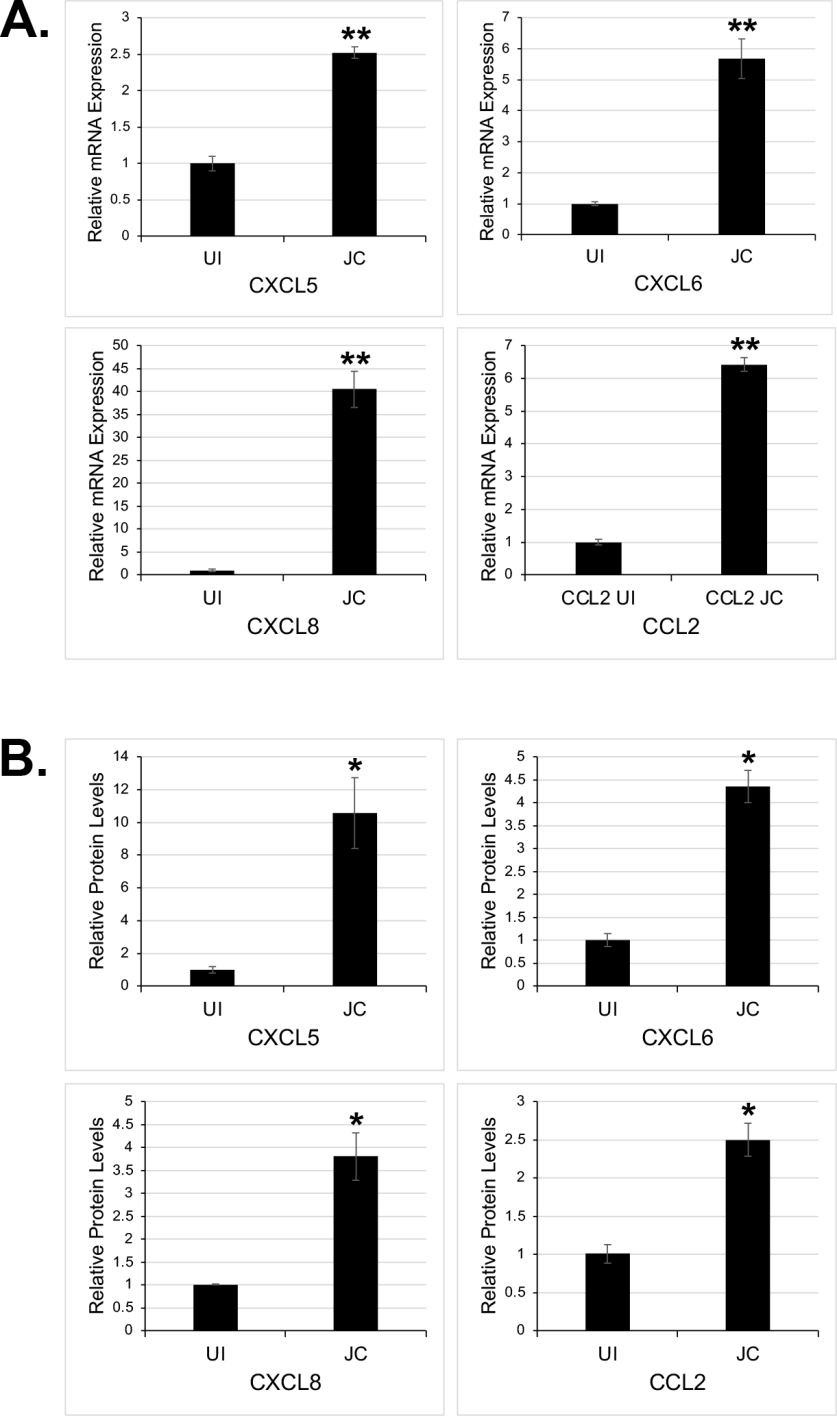
JCPyV infection triggers the upregulation of proinflammatory chemokines CXCL5, CXCL6, CXCL8, and CCL2 in CPE cells. (**A**) RT-qPCR of chemokine mRNA expression compared to uninfected control is expressed as fold change over the control. The data represent three independent experiments performed in triplicate. (**B**) Cytokine immunoarrays were used to measure the secretion of chemokines into the supernatant by JCPyV infected cells compared to uninfected cells. The data represent two independent experiments performed in duplicate. Error bars represent standard deviations (SD). **, *P* < 0.01; *, *P* < 0.05.

GO pathway analysis revealed enrichment with proteins involved in proinflammatory responses (Fig. 2A). In particular, a group of genes that were upregulated by JCPyV infection were inflammatory chemokines including C-C motif ligand 2 (CCL2; also known as MCP-1), and the chemokine C-X-C motif ligands (CXCL) CXCL5, CXCL6, and CXCL8 (also known as IL-8) (Fig. 1A and 2B). These results were validated by RT-qPCR using the same RNA as used for the microarray (Fig. 3A). Furthermore, we assayed by immunoarray the secretion of these chemokines (7 d.p.i) and observed an increase of cytokine proteins secreted into the supernatants of infected choroid plexus epithelial cells compared to uninfected controls (Fig. 3B).

**Fig 4:**
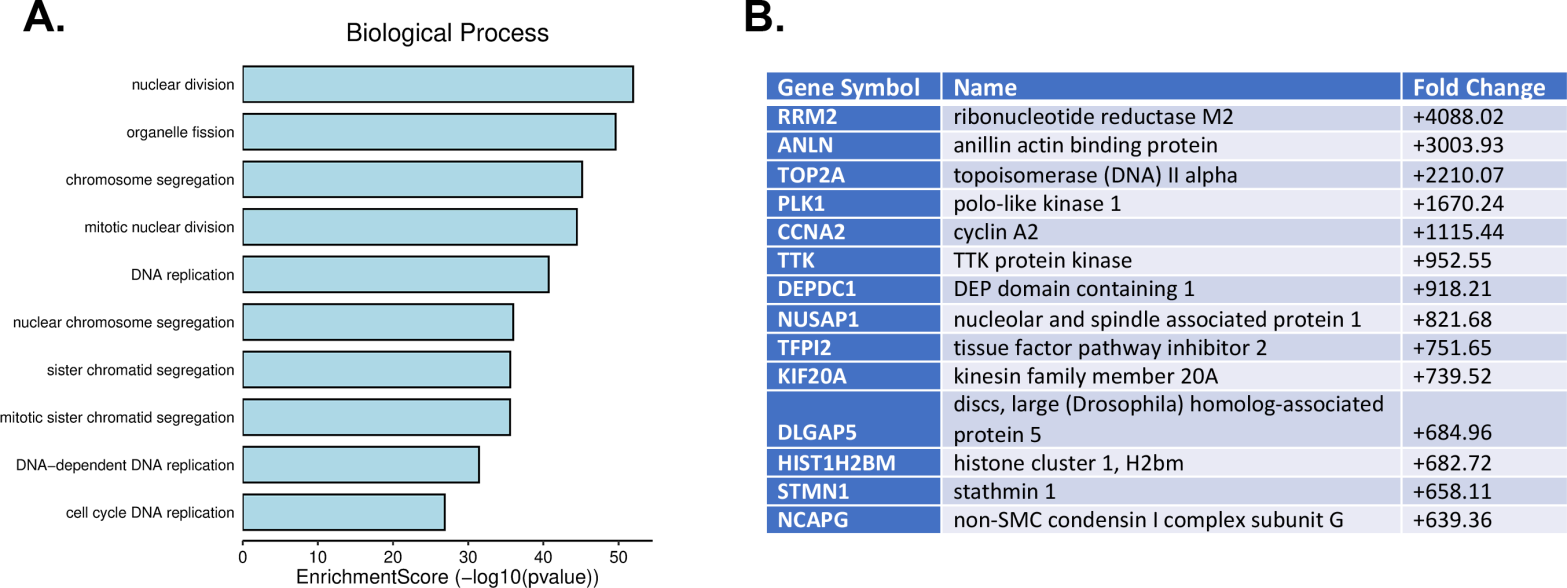
JCPyV infection stimulates expression of genes that regulate the cell cycle and DNA replication. (**A**) GO term analysis genes upregulated by JCPyV infection. (**B**) Fold changes of highly upregulated genes associated with the regulation of biological functions in panel **A**.

Another set of genes significantly affected by infection are involved in the regulation of nuclear division, chromosome segregation, and DNA replication (Fig. 4A and B). This was not surprising, as infection of cells by polyomaviruses induces cells to enter S phase and replicate host cell and viral DNA. These data are consistent with what others have observed studying induction of gene expression in cells infected by JCPyV (25–27).

### JCPyV infection induces the downregulation of tight junction proteins

Some of the most intriguing hits from this screen were in genes coding for the junctional proteins Claudin-1 and Zona occludens 1 (ZO-1, also known as TJP1; Fig. 1A, 5A and B). These two proteins have been shown to be critical for blood-CSF barrier integrity. We confirmed that both of these genes were significantly downregulated in infected cells at seven days post-infection compared to non-infected controls by RT-qPCR and by Western blot (Fig. 6A through C).

**Fig 5:**
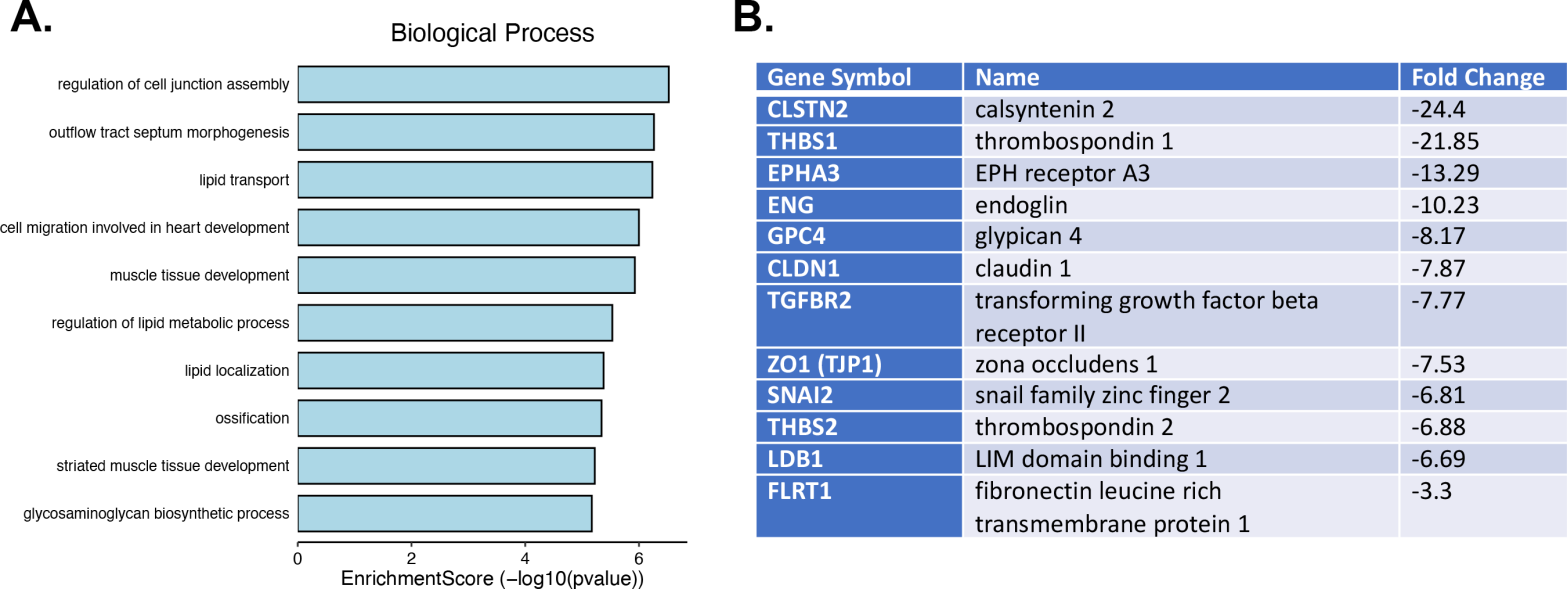
CPEP cells infected by JCPyV downregulate genes involved in barrier integrity. (**A**) GO term analysis of downregulated genes in JCPyV infected cells. (**B**) Fold changes of significantly downregulated genes related to cellular junctions and adhesion.

**Fig 6:**
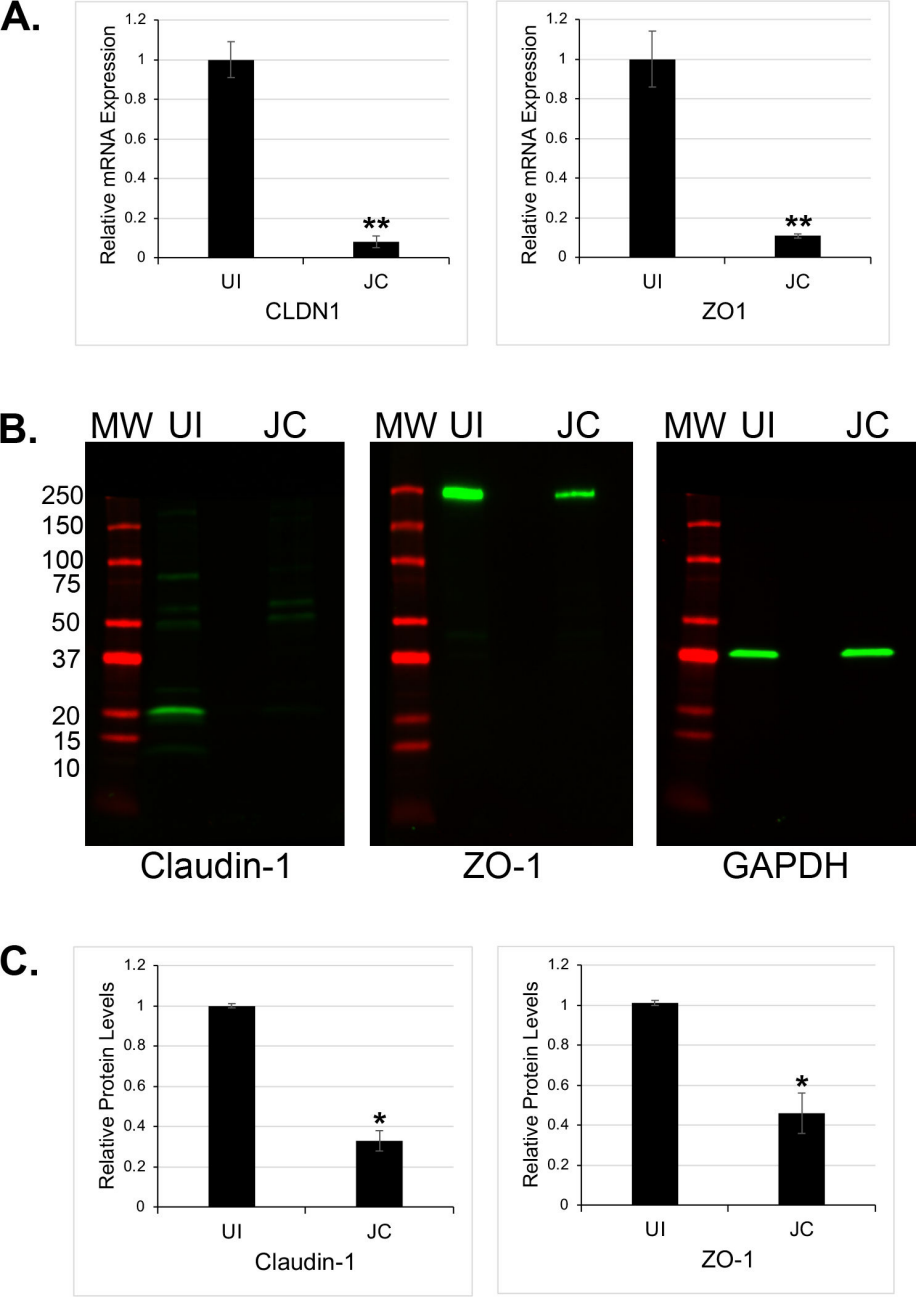
JCPyV induces the downregulation of tight junction proteins. (**A**) RT-qPCR validation of the down-regulation of CLDN1 and ZO1 in infected CPE. The data represent three independent experiments performed in triplicate. (**B**) A representative western blot analyses showing Claudin-1 and zona occludens (ZO-1) protein expression in uninfected (UI) and JCPyV infected (JC) primary CPE. (**C**) Downregulation of tight junction proteins was estimated by densitometric analysis. The data represent three independent experiments performed in triplicate. Error bars represent standard deviations (SD). **, *P* < 0.01; *, *P* < 0.05.

## DISCUSSION

Our data suggest that JCPyV infection of the choroid plexus could directly lead to breakdown of the blood-CSF barrier and indirectly to breakdown of the blood-brain barrier by secretion of inflammatory chemokines. The choroid plexus normally functions as an immune cell reservoir that serves as a specialized site of immune surveillance of the CNS (28). The capillaries in the core of the choroid plexus are fenestrated, and allow immune cells to readily enter the stroma from the peripheral blood. In the stroma of the normal choroid plexus there are resident immune cells, including macrophages, dendritic cells, antigen presenting cells (APCs), and T cells (14, 29, 30). The tight junctions of the choroid plexus epithelium limit the movement of immune cells, molecules, ions, fluids and pathogens into and out of the CNS. However, under proinflammatory conditions resulting from infection, autoimmune diseases, and traumatic brain injury, the choroid plexus has been shown to be an important site from which immune cells infiltrate the CNS (16, 17, 31–34). In experimental autoimmune encephalomyelitis (EAE, a mouse model for multiple sclerosis), CD4+ and CD8+ T cells accumulate in the choroid plexus and enter the CSF during neuroinflammation. Chemokines have been shown to be involved in regulating T cell entry from the choroid plexus into the CNS (35, 36). In addition to animal models, *in vitro* studies using human cells showed that choroid plexus epithelium can regulate the trafficking of T cells into the CSF (37).

Our data show that JCPyV-infected choroid plexus epithelial cells at 7 days post-infection, which represents two full viral life cycles, secrete a signature of proinflammatory chemokines, including CXCL8, CXCL5, CXCL6, and CCL2. These chemokines are known to attract immune cells to sites of infection, including neutrophils, eosinophils, basophils, monocytes, T cells, dendritic cells, and NK cells from the peripheral blood and regulate entry into the CSF from the choroid plexus (38). Increased chemokine expression has previously been associated with polyomavirus infection. A study of the CSF of PML patients found that increased levels of CCL2 was found in PML patients compared to controls, and associated with better prognosis and lower JCPyV levels (39). As CCL2 was markedly upregulated following JCPyV infection of choroid plexus epithelium cells, it is possible that choroid plexus epithelium is the source of this chemokine in the CSF. Interestingly, both CXCL8 and CCL2 expression are downregulated in human glial cells that are resistant to JCPyV infection suggesting that these chemokines could have a stimulatory effect on viral replication (40). Merkel cell polyomavirus infection of fibroblasts induces chemokine expression including CXCL8 in a large T antigen dependent manner (41). In a study of urine samples from patients with BK polyomavirus associated nephropathy (PVAN), CCL2 was identified as a biomarker of disease (42). Additional chemokines, including CXCL10, have also been identified as a urinary biomarker for PVAN (43).

Bacterial pathogens are also known to infect choroid plexus epithelium and elicit a chemokine response. Infection of an adult choroid plexus epithelial cell line (HIBCPP) with Neisseria meningitidis resulted in the upregulation of a number of chemokines including CXCL6, CXCL8, and CCL2 (44). Infection with *Borrelia burgdorferi* induces a set of chemokines including CCL2 (45). Also, in a porcine choroid plexus model of neuroinvasion, *Streptococcus suis* induced chemokine upregulation including CCL2, CXCL5, and CXCL8 (46).

We also observed that JCPyV infection of human choroid plexus epithelial cells leads to the down regulation of proteins critical for the formation of the blood-CSF barrier. Other neuroinvasive pathogens have been shown to disrupt the tight junction proteins of the BCSFB following infection, including echovirus 30 (EV30) (47) and severe acute respiratory syndrome corona virus 2 (SARS CoV2) (48, 49). There is also evidence that chemokines secreted by the choroid plexus epithelium have a role in disrupting tight junction formation. Experiments in a mouse model showed that poly I:C treatment triggered CCL2 secretion by choroid plexus epithelial cells, leading to Cldn1 gene downregulation, disruption of the BCSFB, and subsequent immune cell entry into the CNS (50). In addition, analysis of mouse models looking at injection of LPS resulted in choroid plexus epithelium secretion of chemokines including CCL2 and CXCL5 and the disruption and mislocalization of tight junction proteins (51, 52). *B. burgdorferi* infection of human choroid plexus epithelial cells results in the downregulation of tight junction and adherens junction proteins (45). Infection of porcine choroid plexus epithelial cells *S. suis* resulted in Claudin-1 and ZO-1 rearrangement, disorganization, and barrier disruption (53). The loss of these tight junction proteins, Claudin-1 and ZO-1, and increased chemokine secretion by JCPyV infection may lead to disruption of barrier function in both the BCSFB and BBB.

The choroid plexus epithelium has been shown to be directly infected by a variety of other neuropathogenic viruses, including EV30, SARS CoV2, and herpes simplex virus 1 (HSV-1) (18, 54). Following infection by EV30, the choroid plexus epithelial cells secrete a set of chemokines, including CXCL8 (IL-8) (55). CXCL8 levels were found to be increased in the CSF of EV30 meningitis patients (56). Subsequent EV30 of the choroid plexus epithelial cells leads to the infiltration of T cells (55, 57). HSV-1 has been shown to infect the choroid plexus in cases of human neonatal encephalitis (58). Recently, SARS CoV2 infection of choroid plexus epithelium has been described in clinical samples as well as human organoid studies. SARS CoV2-infected choroid plexus epithelium secretes proinflammatory chemokines, including CXCL8 and CCL2. Increased CXCL8 is found in the CSF of patients infected with COVID-19 (59). Studies of choroid plexus from SARS patient samples, the choroid plexus epithelial cells display proinflammatory chemokine secretion, followed by entry of T cells into the brain parenchyma (60), and there is evidence of direct SARS CoV2 infection of choroid plexus epithelium in human brain tissue samples (61). In addition, productive SARS CoV2 infection of choroid plexus epithelium in an organoid model system has been shown to increase the secretion of both CCL2 and CXCL8 (49).

In summary, we have shown that JCPyV infection of primary choroid plexus epithelial cells, similar to other neuroinvasive pathogens, induces a pro-inflammatory response and subsequent secretion of pro-inflammatory cytokines and chemokines. Infection also reduces expression of critical junctional proteins that maintain the blood-choroid plexus barrier. These data have implications for understanding not only how JCPyV invades the CNS but also how immune cells are marshalled to control the infection.

## MATERIALS AND METHODS

### Cells and virus infection

Human choroid plexus epithelial cells at passage 3 (ScienCell Research Labs, catalog #1310) were cultured in cell line-specific complete media, as indicated by the manufacturer in a humidified incubator at 37°C with 5% CO_2_ as previously described (22, 24). Cells were plated at 10,000 cells/cm^2^ in poly-l-lysine-coated in six total T150 flasks. The Mad1/SVEdelta strain of JCPyV was propagated in SVGA cells and purified using cesium chloride as previously described (62–64). For the infected samples (*n* = 3 flasks), cells were grown to 80% confluency and then challenged in serum-free media with cesium chloride-purified JCPyV for 1 hour at 37°C. Uninfected control cells (*n* = 3 flasks) were treated exactly the same in the absence of virus. Virus-containing media were then aspirated and infected cultures were maintained for 7 days.

### RNA isolation, transcriptome analysis, and validation

Following infection and subsequent incubation, cells were collected using Cellstripper (Corning Inc.), and washed twice in PBS, with an average of 7 million cells counted per flask. Total RNA was isolated using the RNeasy Plus Mini Kit (Qiagen, catalog #74134) according to the manufacturer’s instructions.

RNA purity and quality were verified with Nanodrop and BioAnalyzer measurements. Biotin-labeled cDNA was produced from 100 ng of total cellular RNA and hybridized to the Affymetrix Human Clariom D Microarrays (ThermoFisher Scientific, Catalog #902914) according to the manufacturer’s instructions. The arrays were scanned using an Affymetrix GeneChip Scanner to obtain .cel files. The raw microarray CEL data files were normalized and analyzed with Transcriptome Analysis Console software to produce fold change values (ThermoFisher Scientific, Version 4.0). Genes with a fold change of >2 or <−2 and a false discovery rate (FDR) of *P* < 0.05 were considered differentially regulated. Pathway analysis was performed using the Ingenuity Pathway Analysis (IPA; Qiagen) and SRPlot (65).

Real time PCR was performed using the Bio Rad iScript Reverse Transcription Supermix for RT-qPCR (Bio-Rad, Catalog #1708891) and TaqMan Fast Advanced Master Mix (Applied biosystems, Catalog #4444556). TaqMan Gene Expression Assays were obtained from ThermoFisher Scientific. RT-qPCR was performed with a BioRad CFX96 detection system. Expression levels of genes of interest were normalized to GAPDH as an internal control. Each reaction was performed in triplicate in three independent experiments.

### Western blots

Samples of uninfected and infected (JCPyV, 7 d.p.i.) CPEP cells were lysed on ice for 30 minutes in RIPA (radioimmunoprecipitation assay buffer; Thermo, 89900) supplemented with complete EDTA-free protease inhibitor cocktail (Sigma/Roche,11836170001) and 0.1 mM PMSF, sonicated, and centrifuged at 10,000 RPM at 4°C for 20 minutes to pellet debris. Protein content was determined using the Pierce Micro BCA Protein Assay kit (Thermo, 23235). Samples were prepared in 4 x loading dye (Bio-Rad Laboratories, Hercules, CA) and 15% BME, boiled at 95°C for 10 min, and loaded in 4%–15% gradient Mini-Protean TGX Stain-Free precast gels (Bio-Rad). Gels were run at 120V and transferred to a 0.2 µm nitrocellulose membrane by the semidry transfer method. Blots were blocked in 1% BSA/Tris-buffered saline with 0.01% Tween 20 (TBST) for 1 hour at room temperature. Primary antibodies (GAPDH, CST5174, 1:1,000; Claudin 1, Thermo PA5-16833, 1:500; ZO-1/TJP1, CST13663, 1:500) were diluted in blocking buffer and incubated overnight at 4°C. Blots were washed three times with TBST and incubated with secondary antibody diluted in TBST for 1 hour at room temperature. The blots were then incubated with fluorescently labeled secondary antibodies (Thermo A32735, 1:5,000) diluted at 1:5,000. Blots were washed three times with TBST and then imaged on a ChemiDoc MP imaging system and densitometry was quantified using Image Lab software (BioRad). All western blots and densitometry analysis were performed in triplicate.

### Immunoarray

To evaluate the effect of JCPyV infection on chemokine secretion, human cytokine/chemokine array membranes (RayBiotech, Inc., catalog #AAH-CYT-1000-8) were used according to the manufacturer’s instructions. Briefly, membranes were blocked for 30 minutes at room temperature, then undiluted supernatants from uninfected and infected (JCPyV, 7 d.p.i.) CPEP cells were added to the membranes and incubated overnight at 4°C. The following day the supernatants were removed and membranes washed and then incubated with biotinylated anti-chemokine antibodies overnight at 4°C. Membranes were then washed, incubated with streptavidin conjugated to HRP for 4 hours at room temperature, washed and samples were detected by chemiluminescence. Immunoarray experiments were performed in duplicate in two separate experiments. Images were acquired using a ChemiDoc MP System (BioRad) and densitometry was quantified using Image Lab software (BioRad).

### Statistical analysis

Statistical analyses were performed using Microsoft Excel and GraphPad Prism version 10 (GraphPad Software, Inc.). Statistical results are presented as the mean ± standard deviation (SD). Student’s *t*-test was used to calculate the significance of differences between pairs of samples as indicated. *P* values < 0.05 were considered statistically significant and indicated as follows: **, *P <* 0.01; *, *P <* 0.05.

## Data Availability

The microarray data were deposited into the Gene Expression Omnibus (GEO) database (GSE264236).
